# Do Topical Repellents Divert Mosquitoes within a Community? – Health Equity Implications of Topical Repellents as a Mosquito Bite Prevention Tool

**DOI:** 10.1371/journal.pone.0084875

**Published:** 2013-12-20

**Authors:** Marta Ferreira Maia, Sangoro Peter Onyango, Max Thele, Emmanuel Titus Simfukwe, Elizabeth Louise Turner, Sarah Jane Moore

**Affiliations:** 1 Swiss Tropical & Public Health Institute, Department of Epidemiology & Public Health, Basel, Switzerland; 2 University of Basel, Basel, Switzerland; 3 Ifakara Health Institute, Environmental Health and Ecological Sciences Thematic Group, Bagamoyo, Pwani Region, United Republic of Tanzania; 4 London School of Hygiene & Tropical Medicine, Department of Disease Control, London, England, United Kingdom; 5 Duke University, Department of Biostatistics and Bioinformatics, Durham, North Carolina, United States of America; 6 Duke University, Duke Global Health Institute, Durham, North Carolina, United States of America; University of Crete, Greece

## Abstract

**Objectives:**

Repellents do not kill mosquitoes - they simply reduce human-vector contact. Thus it is possible that individuals who do not use repellents but dwell close to repellent users experience more bites than otherwise. The objective of this study was to measure if diversion occurs from households that use repellents to those that do not use repellents.

**Methods:**

The study was performed in three Tanzanian villages using 15%-DEET and placebo lotions. All households were given LLINs. Three coverage scenarios were investigated: complete coverage (all households were given 15%-DEET), incomplete coverage (80% of households were given 15%-DEET and 20% placebo) and no coverage (all households were given placebo). A crossover study design was used and coverage scenarios were rotated weekly over a period of ten weeks. The placebo lotion was randomly allocated to households in the incomplete coverage scenario. The level of compliance was reported to be close to 100%. Mosquito densities were measured through aspiration of resting mosquitoes. Data were analysed using negative binomial regression models.

**Findings:**

Repellent-users had consistently fewer mosquitoes in their dwellings. In villages where everybody had been given 15%-DEET, resting mosquito densities were fewer than half that of households in the no coverage scenario (Incidence Rate Ratio [IRR]=0.39 (95% confidence interval [CI]: 0.25-0.60); p<0.001). Placebo-users living in a village where 80% of the households used 15%-DEET were likely to have over four-times more mosquitoes (IRR=4.17; 95% CI: 3.08-5.65; p<0.001) resting in their dwellings in comparison to households in a village where nobody uses repellent.

**Conclusions:**

There is evidence that high coverage of repellent use could significantly reduce man-vector contact but with incomplete coverage evidence suggests that mosquitoes are diverted from households that use repellent to those that do not. Therefore, if repellents are to be considered for vector control, strategies to maximise coverage are required.

## Introduction

'Topical repellents like N,N-Diethyl-meta-toluamide (DEET ) or para-menthane-3,8-diol (PMD) are excellent tools to protect individuals against mosquito bites especially in areas where vectors usually bite early in the evening [1-4].These compounds interfere with the mosquito’s olfactory system preventing them from identifying their hosts [5] and obtaining a blood-meal [6]. However, repellents do not kill the host-seeking mosquitoes, they simply reduce man-vector contact. In order to be effective, repellents must be applied every evening and regular compliance is essential. 

Repellents may play an important role in public health, as they are one of the few tools that can protect against mosquitoes that feed outside sleeping hours, during which time long-lasting insecticidal nets (LLINs) are available and effective. To date, vector control programs in sub-Saharan Africa have focused on the implementation of strictly intra-domiciliary interventions like LLINs and indoor residual spraying (IRS). These interventions focus on reducing human-vector contact indoors and have contributed to a malaria decline in various regions of sub-Saharan Africa [7]. However, the use of LLINS cause selective pressure upon mosquito populations, because those interventions target only indoor and late-evening biting mosquitoes, resulting in an increased relative abundance of early-evening biting disease vectors that are not killed by these interventions [8]. In addition, urbanization and *and the greater access to electricity* gives many people a reason to stay awake later, increasing the availability of humans for early feeding and vector borne disease transmission [9]. 

In southern Tanzania, despite high bed net coverage, malaria transmission persists. It is estimated that around 20% of transmission occurs before individuals retire to bed, therefore topical repellents may have a benefit if used as an additional vector control tool to LLINs in this setting [10]. However, there are several problems related to implementation of topical repellents: assuring compliance and reaching complete coverage. If a proportion of the population at risk does not use repellents there is a chance that mosquitoes will be diverted to non-repellent users [11]. In this case people who are not using repellents as a result of poor awareness or poverty will be more exposed to mosquito bites and consequently to vector borne diseases. Diversion has been observed in households with incomplete bed net coverage, and in some scenarios mosquitoes may feed on animals, which would enhance protective efficacy [12]. In the Gambia a study using permethrin-impregnated bed-nets observed that despite mosquitoes being deterred from entering experimental huts by the volatile chemicals, no increase in mosquito numbers were observed in neighbouring huts [13]. It is therefore crucial to investigate neighbouring effects and evaluate if mosquito diversion occurs between households in a community of repellent users and non-users to ensure health equity if interventions with a repellent mode of action are to be considered as public health interventions. Therefore, the present study was devised to measure if diversion occurs from households that use repellents to those that do not within a community with 80% topical repellent coverage.

## Methods

### Ethics statement

Ethical approval was obtained from the Ifakara Institutional Review Board (IHI-IRB) Certificate number: IHRDC/IRB/A46 and from the Tanzanian National Institute for Medical Research (NIMR) Certificate number: NIMR/HQ/R.8a/Vol. IX/780. All villagers were invited to several community meetings held by the village heads and the research team where the outline of the study and their participating role was explained in the local language. Enrolment was conducted after the meetings and only on signed written informed consent. Participants were given information of mosquito bite prevention using LLINs, covering with clothing and using repellents. A medical officer was employed by the project to offer free health assistance to all participants during the study period. When the study was completed, the research team provided each community with a local, deep-water well to improve access to clean water.

### Study area

The study was conducted for ten weeks from August to October 2010, in Mbingu village, which is approximately 40 km west of Ifakara at 8.21°S and 36.24°E, in Kilombero Valley, in South-Eastern Tanzania. The site is characterized by typical rural houses surrounded by rice and banana fields close to the Londo River and the slopes of the Udzungwa Mountains. The main malaria vectors are *Anopheles gambiae* s.s and *Anopheles arabiensis*. The most common culicines are *Culex quinquefasciatus*, *Mansonia africanus*, *Mansonia uniformis, Culex univattus*, *Aedimorphus ochraceus* and *Coquillettidea aureus.*

### Study participants

All existing 49 households of three sub-villages, Matete, Upper Sanje and Lower Sanje, were recruited, which comprised 16, 13 and 20 households, respectively (Table 1). The villages were at least 500m apart and separated by tall, dense maize vegetation, which reduced the likelihood of mosquitoes moving between villages. All residents over 6-months of age were enrolled upon written informed consent, where parental consent was obtained for children under the age of 18. Withdrawal of participation was permitted at any time. A total of 232 individuals were enrolled in the study, and nobody in the villages refused to participate in the project. The median number of household members was 4 individuals (Interquartile range [IQR]= 2-5). Each household was given one double-size Olyset long-lasting insecticide treated net per sleeping space before the start of the study. There was no withdrawal of study participants throughout the project.

**Table 1:** Characteristics of the 49 study households from three village hamlets in Mbingu, Southeastern Tanzania.

Characteristic		Matete (N=16)	Lower Sanje (N=13)	Upper Sanje (N=20)
Number of occupants	1-3	6 (37.5)	5 (38)	8(40)
	4-6	6 (37.5)	7 (54)	10 (50)
	>6	4 (25)	1 (8)	2 (10)
Household type	Mud walls	7 (44)	(0)	9 (45)
	Burnt brick walls	6 (37)	13 (100)	11 (55)
	Thatch walls	3 (19)	0 (0)	0 (0)
Roof type	Thatch roof	15 (94)	6 (46)	15 (75)
	Metal sheets	1 (6)	7 (54)	5 (25)
Eaves	Present	16 (100)	13 (100)	20 (100)
Households with under 5’s		10 (63)	9 (69)	12 (60)
Households using ITNs		16 (100)	13 (100)	20 (100)
Households with livestock	Chickens	9 (56)	9 (69)	12 (60)
	Goats	0 (0)	0 (0)	1 (5)
	Pigs	0 (0)	1 (8)	0 (0)

Numbers in parentheses are percentages of total number of households per village.

### Study design

The study was designed to test if mosquitoes are diverted from households that use repellent to those that do not in a rural Tanzanian setting. A crossover design was used and coverage scenarios were rotated on a weekly basis for a period of 10 weeks (Table 2). The design was balanced on treatment, which was allocated at household level, i.e DEET-15% or placebo. Each household was allocated each treatment, independent of the coverage scenario they represented, an average of 5 times. Three coverage scenarios were investigated: 1) Complete topical repellent coverage where all households in a village were given 15% DEET; 2) No coverage where all households in a village were given a placebo and; 3) Incomplete topical repellent coverage where 80% of the households were given DEET-15% and the remaining 20% were given a placebo. During the 10-week study period, complete repellent coverage was allocated 3 times; no coverage scenario was allocated 10 times; and incomplete coverage scenario was allocated 17 times (Table 2). This was done to ensure balance of treatment allocation, compensating for the low number (20%) of placebo households in the incomplete coverage scenario where diversion could be measured. Complete coverage was only measured in two villages.

**Table 2:** Rotation of the treatment scenarios over 10 weeks sampling period in Matete, Upper Sanje and Upper Sanje.

**Week**	**Matete**	**Upper Sanje**	**Lower Sanje**
**1**	100% Coverage	No coverage	80% coverage
**2**	100% Coverage	No coverage	80% coverage
**3**	No coverage	100% Coverage	80% coverage
**4**	80% coverage	80% coverage	No coverage
**5**	80% coverage	80% coverage	No coverage
**6**	80% coverage	No coverage	80% coverage
**7**	No coverage	80% coverage	80% coverage
**8**	80% coverage	80% coverage	No coverage
**9**	80% coverage	No coverage	80% coverage
**10**	No coverage	80% coverage	80% coverage

The treatment allocation of placebo or DEET 15% was fully randomised within the 80% coverage, however no household was allocated placebo in consecutive weeks of the 80% coverage and each house was at least once included in the placebo group of the 80% coveage. Lower Sanje intentionally did not receive the 100% coverage scenario in order to allow the study to be balanced on the number of times a household received placebo or DEET 15%.

The households of the village allocated to the incomplete coverage scenario were selected using restricted randomisation with a lottery method to DEET-15% or placebo in the ratio of 80% to 20% respectively. No household was allocated placebo in consecutive weeks of the incomplete coverage scenario to prevent repeated sampling of the same households with the same intervention. This was achieved by ensuring that when the incomplete coverage scenario was repeated in consecutive weeks in a village, all placebo households were allocated to treatment in the consecutive week. 

To blind both the households and field team to the type of lotion received, the 15%-DEET lotion and placebo lotion were provided in identical bottles. The bottles were identifiable to the investigator by a 3-digit code. Compliance was inspected on a weekly basis through measuring the weight of the repellent and placebo bottles that had been distributed. All bottles were collected at the end of each week and were replaced with new bottles of the appropriate lotion for the following week. 

### Mosquito collection

**Figure 1:**
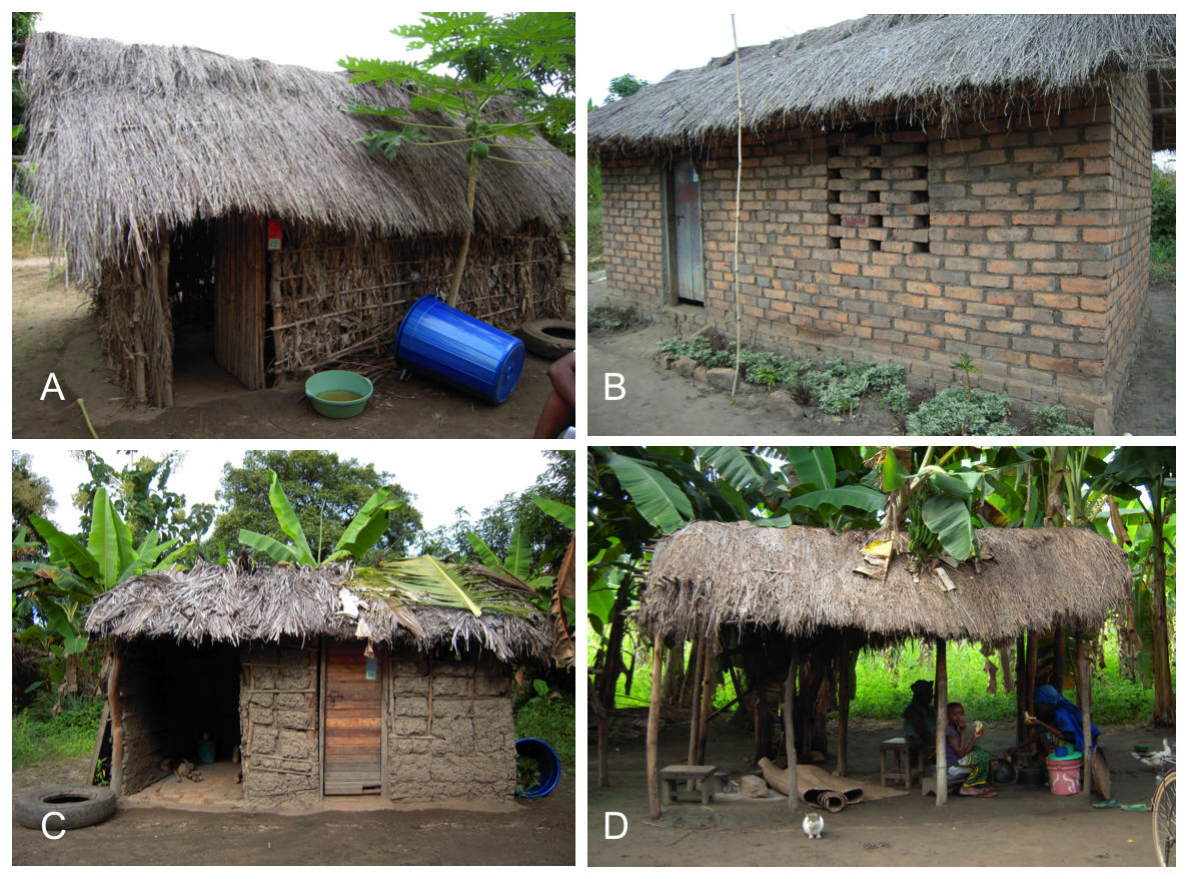
Mosquito collection sites and some households of the Mbingu area, Kilombero Valley, Tanzania. A – Household made with thatch wall and thatch roof with a blue barrel and a car tyre serving as artificial resting places. B – Household made with brick walls and thatch roof, the artificial resting places are not visible because they were deployed behind the household. C – Household made of mud walls and thatch roof with a blue barrel and a car tyre serving as artificial resting places. D – *Kibanda*, outdoor kitchen area.

Weekly mosquito collections were performed on three consecutive weekdays where each household was sampled once. Typically, each village was visited on one day each week. Host seeking mosquito densities from the enrolled households were estimated through the number of resting mosquitoes collected from early morning indoor and outdoor aspirations using aspirating devices [14]. Outdoor resting mosquitoes were sampled from artificial resting places immediately next to each household and from underneath the thatch roof of the outdoor kitchen area of each household (Figure 1). The artificial resting shelters, installed by the research team, consisted of a large dark barrel covered with vegetation (diameter 0.65m and depth 0.75m) and a regular car tyre placed directly outside each household. Collections were done indoors and outdoors to maximize the number of collected resting mosquitoes per household. After collection, mosquitoes were sorted by genera and members of the *Anopheles gambiae* complex were identified using PCR (polymerase chain reaction) [15].

### Statistical analysis

Analysis was performed at the household level using a generalized linear model (GLM) fitted using STATA 11.0 statistical software (College Station, TX: StataCorp LP). The aggregated number of resting mosquitoes aspirated indoors and outdoors at each household was the dependent variable. The number of mosquitoes collected indoors and outdoors was aggregated because we were interested in the household mosquito density and not in differences in resting densities. A negative binomial distribution was assumed to account for over-dispersion of the daily mosquito count data. The generalized estimating equations (GEE) approach was used to account for the non-independence of mosquito counts for a given household since each household was measured each week of the study. We assumed an exchangeable correlation matrix. The log link was used and robust standard errors were estimated to allow for model mis-specification. Coverage scenario was modelled using a set of 3 indicator variables, where no coverage was considered the reference at a household level. The model was used to derive incidence rate ratios (IRR) of resting mosquito densities in households per day in each of the three scenarios (complete coverage and each of a repellent-using and non-repellent using household in the 80% coverage intervention) compared to no coverage as reference. Fixed effects for village and week were included in all models to account for the study design. Additional sources of variation considered were: day of week of mosquito collection, previous week’s treatment (to account for possible carry-over effects), household structure, presence of open eaves, number of occupants and individual mosquito collectors. Significance testing was performed at the 5% level. 

## Results

Of the 49 households enrolled in the study, we collected mosquitoes from 44 households at each of the 10 weekly visit.. Of the remaining households, 3 households were missing one measurement and two other households were missing 2 and 4 measurements, respectively. Overall, 481 household-days of mosquito data were available for analysis. A total number of 2650 resting mosquitoes were collected, of which 105 were anophelines (83% were *Anopheles gambiae* s.s. and the remaining *Anopheles arabiensis*). Most mosquitoes were found resting indoors (47.6%), followed by underneath the thatch roof of the outdoor cooking areas (*kibanda*) (20.4%), from inside the large barrel (17.9%) and from inside the car tyre (14.1%) (Table 3). Most of female mosquitoes collected were unfed (Table 4). Of the 49 households in the study, 33% had mud walls and 61% had burned mud brick walls; 73% were thatched with grass and 27% had corrugated steel roofs (Table 1). A few households (61%) owned chickens; no households owned cattle. There were considerable differences in housing structure between the three villages with Matete having poorer housing, a factor that might influence mosquito densities inside houses. However, there was no evidence that roof type or wall types were associated with the outcome. Other sources of variance including day of week, number of occupants, previous week’s treatment and collector were shown to be not significant in the model and were therefore excluded from the model. The final model included the pre-specified fixed effects for village and week of the study. Regular spot checks indicated that all project participants complied with the repellent usage.

**Table 3:** Total number of *Anopheles* *gambiae* s.l., *Anopheles funestus* and *Culex* spp. collected resting indoors and outdoors in Matete, Upper Sanje and Lower Sanje with different coverage scenarios.

		***Anopheles gambiae* s.l.**					***Anopheles funestus***					***Culex* spp.**				
		**INDOORS**	**OUTDOORS**				**INDOORS**	**OUTDOORS**				**INDOORS**	**OUTDOORS**			
	**N**	**Total**	**Tyre**	**Barrel**	**Kibanda**	**Total**	**Total**	**Tyre**	**Barrel**	**Kibanda**	**Total**	**Total**	**Tyre**	**Barrel**	**Kibanda**	**Total**
**Matete**																
No coverage	2	**4**	0	7	4	**11**	**2**	0	0	0	**0**	**323**	85	125	78	**288**
100% coverage	3	**0**	0	0	1	**1**	**0**	1	0	0	**1**	**34**	36	29	50	**115**
80% coverage repellent users	5	**8**	2	2	2	**6**	**0**	0	1	0	**1**	**242**	47	80	102	**229**
80% coverage placebo users	5	**1**	0	0	0	**0**	**0**	0	0	1	**1**	**103**	46	35	60	**141**
**Upper Sanje**																
No coverage	1	**1**	0	0	2	**2**	**1**	0	0	0	**0**	**154**	56	87	72	**215**
100% coverage	4	**1**	0	0	0	**0**	**1**	0	0	2	**2**	**22**	4	3	13	**20**
80% coverage repellent users	5	**0**	0	0	0	**0**	**2**	4	3	1	**8**	**53**	7	13	17	**37**
80% coverage placebo users	5	**2**	0	0	2	**2**	**1**	0	0	0	**0**	**49**	13	18	18	**49**
**Lower Sanje**																
No coverage	3	**2**	0	1	0	**1**	**5**	0	1	2	**3**	**16**	3	1	6	**10**
100% coverage	-	**-**	-	-	-	**-**	**-**	-	-	-	**-**	**-**	-	-	-	**-**
80% coverage repellent users	7	**1**	0	0	0	**0**	**14**	1	4	12	**17**	**105**	11	17	42	**70**
80% coverage placebo users	7	**0**	0	0	0	**0**	**1**	0	1	1	**2**	**113**	58	47	52	**157**
**SUB-TOTAL**		**20**	**2**	**10**	**11**	**23**	**27**	**6**	**10**	**19**	**35**	**1214**	**366**	**455**	**510**	**1331**
**TOTAL**		**43**					**62**					**2545**				

Legend: N- number of replicates.

**Table 4:** Total number of mosquitoes in different physiological status by coverage scenarios (N=2650 in total), medians, inter-quartile ranges (IQR), incidence rate ratios (IRR) and 95% confidence intervals (95%CI) of total number of mosquitoes aspirated indoors and outdoors per day per household enrolled in three repellent coverage scenarios: no coverage; complete coverage; households that use repellents in an 80% repellent coverage scenario; and households that do not use repellents in a an 80% coverage scenario from a generalized linear model adjusted for clustering using the generalized estimating approach with adjustment for sub-village and sampling week.

	**N**	***Anopheles gambiae* s.l.**				***Anopheles funestus***				***Culex* spp.**								
		Unfed	Fed	Gravid	**Total**	Unfed	Fed	Gravid	**Total**	Unfed	Fed	Gravid	**Total**	Median	IQR	IRR	95%CI	p-value
No coverage	1038	10 (48)	8 (38)	3 (14)	**21**	4 (36)	4(36)	3(27)	**11**	633 (63)	91 (9)	282 (28)	**1006**	2	[0-9]	1	-	-
100% coverage	197	1 (50)	1 (50)	0 (0)	**2**	1 (25)	2 (20)	1 (25)	**4**	131 (69)	37 (19)	23 (12)	**191**	3	[2-6]	0.39	[0.25 - 0.60]	<0.001
80 % coverage (Repellent users)	776	8 (53)	3 (20)	4 (27)	**15**	14 (33)	12 (29)	16 (38)	**42**	358 (50)	133 (18)	228 (32)	**719**	2	[0-5]	0.92	[0.72 - 1.17]	0.49
80% coverage (Placebo users)	639	1 (20)	4 (80)	0 (0)	**5**	2 (40)	3 (60)	0 (0)	**5**	411 (65)	29 (5)	189 (30)	**629**	8.5	[2-20]	4.17	[3.08 - 5.65]	<0.001

Numbers in parenthesis are the percentage of the total in each coverage scenario.

The lowest densities of resting mosquitoes were found in households within villages where everybody had been given a repellent lotion (Table 4). These households had less than half of the mosquitoes that would normally be found in households in the no coverage scenario (Median=3; IQR=[2-6]; IRR=0.39; 95%CI=[0.25 - 0.6]; p<0.001). Only 8% fewer mosquitoes were found resting in repellent-using households in an incomplete coverage scenario compared to households in a no-coverage scenario (Median=2; IQR=[0-5]; IRR=0.92; 95%CI=[0.72 - 1.17]; p=0.485). On the other hand, placebo households in an incomplete coverage scenario that were surrounded by repellent-using households, had more than 4 times more resting mosquitoes compared to a non-coverage scenario where nobody in a village uses repellent (Median=8.5; IQR=[2-20]; IRR=4.17; 95%CI=[3.07 - 5.65]; p<0.001). Diversion to animals is unlikely to have occurred as none of the households possessed domestic animals besides chickens. 

## Discussion

The WHO recommends topical repellents as the first line of protection against outdoor biting vectors [16]. Similarly, the U.S. Centres for Disease Control and Prevention (CDC) has acknowledged that ‘Repellents are an important tool to assist people in protecting themselves from mosquito- borne diseases’ [17]. Although repellents are popular amongst travellers and are commonly used in some urban areas' [9,18], the local population in rural Tanzania does not commonly use them. This may be because the local population has limited knowledge of repellents; these products are generally unavailable in small local shops in rural areas and are typically not affordable to most of the local population who live on subsistence incomes. Unlike bed-nets and anti-malarial drugs, topical repellents have never been included in subsidization programs in Africa. Countries like South Africa and Thailand recommend their use and some non-governmental organisations (NGOs) provide repellents to populations, including PSI in South America and the Global Fund in the Mekong region of Thailand (Michael MacDonald, personal communication).

The results from this study provide evidence that mosquito diversion from repellent users to non-users occurs under an incomplete coverage scenario. Households of non-repellent users within villages where 80% of the village uses repellents have over four times more mosquitoes resting in their households. We also observed that households that used repellent in an incomplete coverage scenario maintained high numbers of host seeking mosquitoes in their households despite compliance with the intervention. It is unlikely that mosquitoes will feed on these household members since they are protected with mosquito repellent. This is indicative that complete coverage of topical repellents offers a community effect by reducing the number of mosquitoes in all human dwellings more markedly than if only incomplete coverage is reached. Hence complete coverage of topical repellents would not only benefit those who otherwise would not be covered by avoiding diversion of mosquitoes but also individuals who usually comply with topical repellents. Even if topical repellents are made available to all, it is unlikely that complete coverage is attainable since topical repellents require regular compliance. As a result, mosquito diversion is likely to occur between those who comply with the intervention and those that do not. In case of malaria control, complete coverage is the recommended strategy for implementing topical repellents, however, if a large proportion of the population regularly complies with topical repellent usage, it is likely that the malaria parasite load in the community will be reduced through reduction in man-vector contact as has been observed on a community scale with untreated bed nets that act only as a personal protection tool [19]. Our study did not recover many blood fed mosquitoes or malaria vectors due to a drought that occurred during the study. Therefore, it is not clear if malaria vectors are being diverted and feeding on unprotected individuals. We plan future studies that will analyse the blood meal from fed mosquitoes found resting indoors to ascertain whether mosquitoes have fed from humans. Such analyses would help to determine whether diversion results in increased risk of bites to unprotected humans.

Other repellent interventions such as spatial repellents using volatized pyrethroids may offer a better solution than repellents since they prevent mosquitoes from feeding and disrupt their host-seeking behaviour [20]. Such interventions may not necessarily divert mosquitoes to other households but disable the host seeking behaviour of mosquito vectors resulting in a more effective reduction of man vector contact than observed with topical repellents. Currently further research is underway to measure diversion in a community using incomplete coverage of volatized pyrethroids.
